# Countries Response for People With Disabilities During the COVID-19 Pandemic

**DOI:** 10.3389/fresc.2021.796074

**Published:** 2022-02-07

**Authors:** LH Lugo-Agudelo, Maria A Spir Brunal, Ana M Posada Borrero, Kelly M Cruz Sarmiento, Juan C Velasquez Correa, Rosarita Di Dio Castagna Iannini, Manuela Gonzalez Zuluaga, Victor A Ospina, Daniel F Patiño Lugo, Luisa F Mesa Franco, Christoph Gutenbrunner

**Affiliations:** ^1^University of Antioquia, Medellín, Colombia; ^2^Hannover Medical School, Hanover, Germany

**Keywords:** COVID-19, people with disability, rehabilitation needs, disability, SARS-CoV-2

## Abstract

**Background and Objectives:**

During the Coronavirus disease 19 (COVID-19) pandemic, isolation and prevention measures to reduce COVID-19 contagions are essential for the care of all people; these measures should comply with the principles of inclusion and accessibility for people with disabilities (PWD), with all kinds of deficiencies and levels of dependency. Thereby, the aim of this article is to present the measures adopted for PWD or people with rehabilitation needs, for containment, mitigation, or suppression of the SARS-CoV-2 virus in different countries of all continents and of all income levels.

**Methods:**

A narrative approach was used in this article. First, a broad search was carried out in the 193 member states of the UN, and then 98 countries that issued any document, report, or information related to disability and COVID-19 were selected. Finally, 32 countries were included in this article because they presented official information. We considered official sources, the information available in the government, or on the health ministry page of the country. In this way, the countries that presented information which did not correspond to an official source were excluded. The search was conducted in August 2020 and updated in March 2021.

**Results:**

First, the non-pharmacological general interventions for PWD included informative measures and general recommendations during the stay at home, isolation, and biosecurity measures, contagion prevention, detection of positive cases, mobilization measures, and measures implemented in institutions or residences of PWD. Second, we identified the economic and social benefits provided to PWD during the pandemic. Finally, we identified the measures taken by countries according to the type of impairment (visual, hearing, physical, mental, and cardiopulmonary impairment) during the COVID-19 pandemic.

**Conclusion:**

In response to the COVID-19 pandemic, only 50% of countries from the five world regions created and implemented specific measures for PWD to containment, mitigation, or suppression of the SARS-CoV-2 virus. There is very little specific information available about the measures to continue with the care of people with rehabilitation needs and the long-term follow-up of PWD, and for the prevention and response to violence, especially for women with disabilities.

## Introduction

The Coronavirus disease 2019 (COVID-19) pandemic began in the city of Wuhan (China) at the end of 2019 and it was declared, according to the WHO (1), as such in March 2020. With 233,136,147 confirmed cases of COVID-19, including 4,771,408 deaths worldwide (as of September 30, 2021) (2). It drastically changed the priorities of the entire planet, and it made those countries from the five world regions create and adopt isolation and prevention measures to reduce infections in a short time. Furthermore, these measures, which were essential for the care of all people, had to comply with the principles of inclusion and accessibility for all vulnerable population groups.

More than a billion people in the world experience disability nowadays. The current demographic and health shifts are contributing to a rapid increase in the number of people experiencing disability or decline in functioning for substantially larger periods of their lives (3). This number is increasing globally, in part due to aging populations and due to an increase in chronic health conditions (4). Thereby, these trends create increasing demands for health and rehabilitation services, which are very far from being met, particularly in low- and middle-income countries (5).

People with disabilities (PWD) represent a vulnerable population; in this way Centers for Disease Control and Prevention (CDC) and WHO stated that some PWD may be more likely to become infected with the SARS- CoV-2 virus, or may develop a serious illness due to the underlying medical conditions, congregational living environments, systemic social inequities, and some barriers they might face in accessing healthcare during the pandemic (6–8). Thereby, rehabilitation must be an integral part of COVID-19 management, and it must be kept a health priority during the COVID-19 pandemic, and given adequate financial resources (9). Therefore, each country needs to develop specific strategies for PWD to protect its rights.

It is expected that COVID-19 affects this vulnerable population. Too often, PWD is left behind in emergencies, and this is a risk in the ongoing COVID-19 pandemic (7). Pandemics, such as COVID-19, place everyone at risk, but certain risks are differentially more severe for groups already vulnerable by the preexisting forms of social injustice and discrimination (10). For this reason, any response to the pandemic must be bound with the legal standards, principles of distributive justice, societal norms of protecting vulnerable populations, and core commitments of public health, to ensure that established inequities are not exacerbated (11).

In some countries where health services have been accessible and affordable, governments find it increasingly difficult to respond to the growing health needs of populations and the rising costs of health services (12). During the COVID-19 pandemic, rehabilitation services are facing additional challenges. These services have been defined in many settings as “non-essential,” and many of them have been canceled or limited, for instance, by limiting the care to outpatient settings (9, 13, 14). Furthermore, the huge impact of the COVID-19 pandemic left some patients, families, and caregivers alone with their needs. Associations made great efforts to assist their members by offering information, advice, and individual support (15).

In this way, rehabilitation services must keep continuing during the pandemic; it is an essential component of high-value care to optimize physical and cognitive functioning to reduce disability. In this way, the interruption of these services may affect the well-being and quality of life of PWD and impose more burdens on a population that is already vulnerable (9, 16). Some medical conditions, such as stroke, spinal cord injury, and cardiopulmonary conditions can be aggravated by a lack of access to rehabilitation services (9).

One study described the timely innovative proposals of scientific associations and rehabilitation professionals of different countries, focusing on delivering rehabilitation services, protection and prevention measures, physical distancing, isolation, hand washing, and disinfection measures in the context of the COVID- 19 pandemic. In this way, measures to prevent and protect against transmission of COVID-19 are necessary for all patients in rehabilitation care around the world (17).

Bettger (16) published a commentary to describe the adjustments to the continuum of rehabilitation services across 12 low-income, middle-income, and high-income countries in the context of national COVID-19 preparedness responses and to provide recommendations for decision-makers on the provision and payment of these essential services (18). Another study presented information about 38 countries that showed a huge impact on PWD due to a reduction in all rehabilitation activities in Europe in all the settings, such as acute, postacute, and outpatients (14).

This information allows for knowing some of the adaptations and reorganization of the rehabilitation services carried out in different countries to the health emergency by COVID-19, which had an impact on PWD or with rehabilitation needs (17, 18). Until now, it is known about the response to PWDs that some supranational organizations, rehabilitation associations, and some countries have had during the COVID-19 pandemic. However, a recompilation of the measures of different countries of the five regions of the world has not been made up till now.

The main aim of this study was to describe the measures adopted by different countries around the world and of all income levels to guarantee universal coverage, access to information, and actions for prevention and mitigation of direct and indirect consequences of the COVID-19 pandemic, on PWD and with rehabilitation needs.

## Methods

We used a narrative approach (19) to identify countries' responses to PWD and rehabilitation needs during the COVID-19 pandemic.

### Research Question

Which were the responses and measures adopted by different countries for PWD and with rehabilitation needs, for containment, mitigation, or suppression of the SARS-CoV-2 pandemic?

### Search Strategy

#### Selection, Extraction, and Categorization of Information

A narrative approach was used to identify the measures adopted by different countries for PWD or with rehabilitation needs during the COVID-19 pandemic. First, a broad search was carried out in the 193 member states that belong to the UN (20). Then 98 countries that issued any document, report, or document related to disability and COVID-19 were selected.

In this way, the countries that presented information that did not correspond to an official source were excluded. We considered the official sources, the information available in the governments, or the health ministry pages of the country. Thus, we excluded 66 countries that presented information that did not correspond to an official source. We considered the following official sources: (1) The information is available on the government page of the country. (2) The information is available on the ministry page of the country. We excluded other sources of information, such as NGOs, foundations, private organizations, or independent organizations because this was not the focus of this review. The search was conducted in August 2020 and updated in March 2021.

Eight researchers (MAS, KMC, AMP, JCV, LM, RD, MG, and VO) conducted the data extraction using a predefined form to record the following information: (1) date of the declaration of emergency; (2) specific measures for PWD related to social distancing, biosecurity measures, travel restrictions, detection and tracking of cases, isolation of cases of PWD, economic benefits, and others related to the guarantee of rights; and, (3) specific measures according to the type of disability: hearing disability, visual disability, physical disability, cardiopulmonary limitation, intellectual disability, autism, and dementia.

**Figure 1:**
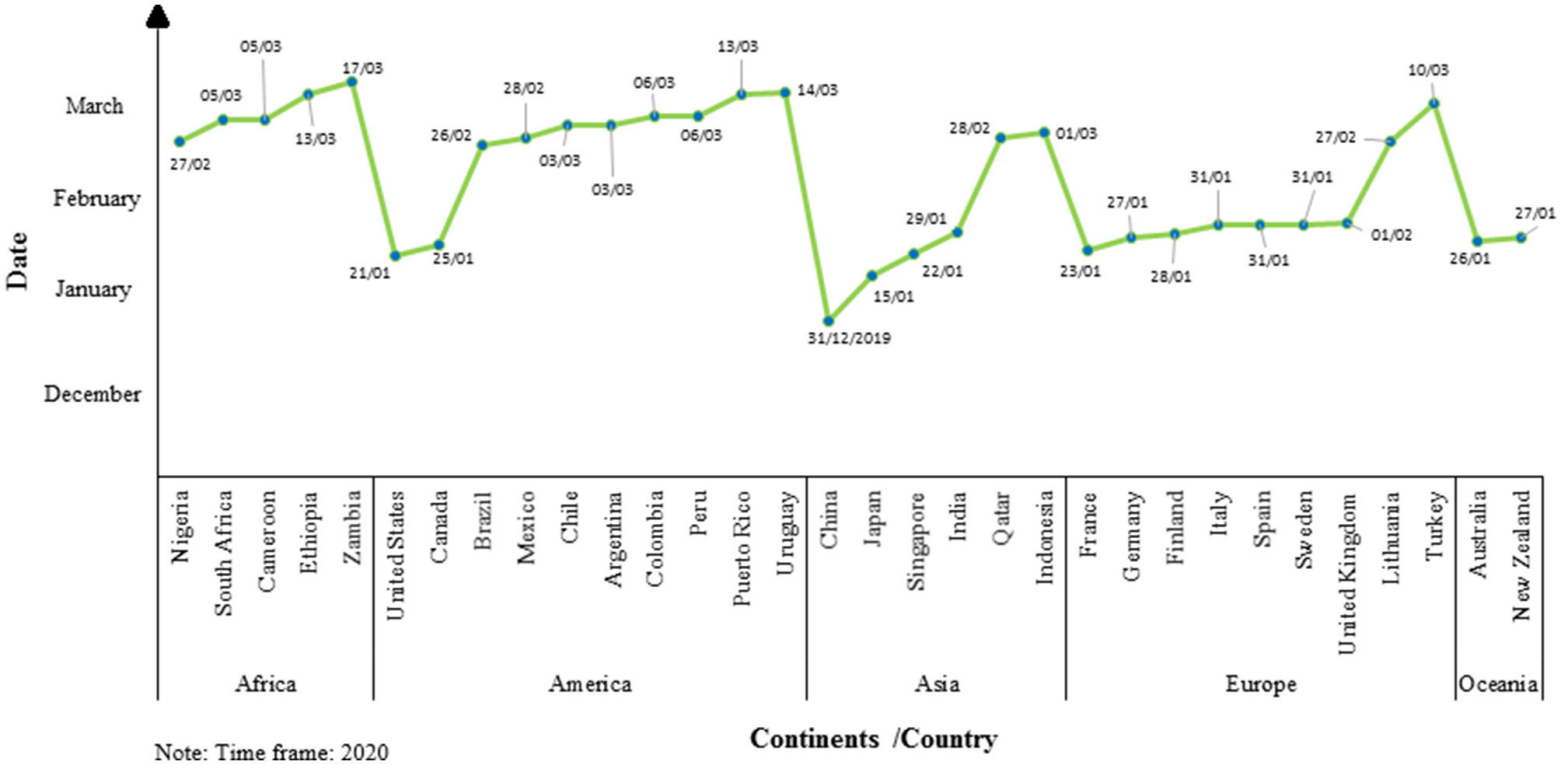
Line graph of the date of declaration of health emergency by COVID-19 according to the countries and continents in the world.

Finally, four researchers with expertise in rehabilitation screened the search results and selected 32 countries from five world regions: Africa (5 countries), America (10 countries), Asia (6 countries), Europe (9 countries), and Oceania (2 countries). Hence, 32 countries were included in this article because they presented official information issued by the government or the health ministry's official pages (refer to Figure 1).

We categorized the information in non-pharmacological interventions for general PWD and interventions according to specific types of disabilities. First, we included the following interventions: informative measures and general recommendations during the lockdown, protection measures and prevention of COVID-19 contagion, detection of positive cases, management, and isolation of PWD; mobility, transport, and isolation; and measures taken within care institutions. Then, we categorized the interventions according to the following types of disability: visual impairments, hearing impairments, physical disability, cardiopulmonary limitation, and mental function that included autism, dementia, and intellectual disability.

## Results

The findings reflect that some governments have made an effort for reducing the transmission of the disease in this vulnerable population because they have recognized the importance of providing access to health services and the need to adopt special and different measures for the prevention of contagion in PWD (21) (refer to Table 1).

**Table 1:** Non-pharmacological general interventions for people with disabilities (PWD).

**Type of measure**	**Description**	**Countries**
Informative measures and general recommendations during the stay at home	Guides, documents, and questions and answers section on health protection with recommendations for social distancing, the requirement to remain accompanied, accommodation plans, permanence at home, minimization of contact, guidance for homes, and health care Special attention during isolation, home assistance for activities of daily living with volunteers, and the permanence of social services Availability of services through telehealth and home care	Mexico (22), United States (6), Spain (23), Canada (24), Singapore (25), United Kingdom (26), France (27) Singapore (28) Lithuania (29) Colombia (21), Uruguay (30), India (31)
Protection measures and prevention of contagion of COVID-19	Measures and recommendations about bioprotection, isolation, prevention of contagion. These were made through guides, documents in accessible formats, didactic sheets, infographics, seminars, informative texts, booklets, and others Measures to help enforcement officers, frontline agencies and employees in supermarket chains to identify and interact with people with disabilities and special needs. This has allowed a greater flexibility in the enforcement of COVID-19-related measures such as the compulsory wearing of facemasks	Colombia (21), Argentina (32, 33), Brazil (34), Uruguay (35), Mexico (36), Perú (37), United States (38), Canada (39), India (40), Indonesia (41), Qatar (42), France (43), Sweden (44), Italy (45), Spain (23), Australia (46), New Zealand (47), South Africa (48) Singapore (17)
Detection of positive cases, management, and isolation of PWD	Documents with information on conducting virus detection tests Prioritize the detection of the COVID-19 virus in the vulnerable population (Disable and old people) Isolation recommendations and steps to follow in case of COVID-19 infection Avoiding the spread of the virus and instructions for home treatment in case of infection Recommendations for staying at home in coexistence with other members in case of COVID- 19 infection Special considerations about isolation. Specific instructions when PWD seek medical attention.	Australia (49), Puerto Rico (50), France (51) Nigeria (52) Singapore (53) Qatar (54) Finland (55) Australia (56, 57) Finland (58)
Mobility, transport, and isolation	Special transport for PWD and their caregivers, to avoid public transport Allowing family members mobility within the national territory to care for the person with a disability Restriction of mobility no more than 500 meters from your residence.	United Kingdom (59) Peru (60) Argentina (61, 62)
Measures were taken in care institutions	Creation of private rooms to avoid contagion and promotion of virtual visits Reduction of crowds in care institutions for the elderly or PWD Restriction of visits to care residences Tips for care in hospital centers	Japan (63) Brazil (64) Germany (65) Chile (66)

### Measures According to the Type of Deficiency During the COVID-19 Pandemic

Some countries made a declaration that PWD could have a greater vulnerability in this pandemic because they are constantly faced with physical barriers in the application of hygiene measures or social distancing. Besides, they require support from other people to carry out their daily activities (16).

In this synthesis, we considered the following impairment conditions: visual, hearing, physical impairments, cardiopulmonary limitations, and alterations in mental function (Tables 2–**6**).

**Table 2:** Measures for people with visual impairment in the COVID-19 pandemic.

**Type of measure**	**Description**	**Countries**
Prevention of contagion of COVID-19	Promoting frequent handwashing, especially after touching surfaces, moving maps, handrails, and other objects. Wash particularly the back of the hand, especially if they used for tracking, locating, or targeting actions Wear long-sleeved clothing that allows the forearm to be used for sensitivity without contamination Cleaning the walking stick when leaving and arriving home	United States (6), Brazil (34), Nigeria (67), Sweden (68), Mexico (69), Colombia (70), Uruguay (71), Canada (24), Qatar (72), South Africa (73) Uruguay (71) United States (74), Mexico (22), Costa Rica (75), Chile (75) United States (6), Mexico (69), Uruguay (71), Chile (75)
Special assistance	Disinfecting the guide dog's harness and leash daily and clean their paws and hair with wet wipes or dry shampoo Caregivers of PWD should offer only shoulder support, avoiding other types of contact Staying with the guide person at the health visit, and the guide person should ensure that information about the pandemic should be provided in an appropriate way for this population	Mexico (69), Uruguay (71) Chile (75), Costa Rica (75), South Africa (73) Canada (24)
Special information for visual impairment	Dissemination of audio content with official information about the news of Coronavirus for the visually impaired Dissemination of information about COVID-19 news through the Braille Writing System. Test for COVID-19 at home	Germany (76), United States (77), Sweden (78), Uruguay (79), Nigeria (67), Mexico (80), Colombia (73), Singapore (25) Mexico (80), Singapore (25), Qatar (72), India (81) Puerto Rico (50)

### People With Visual Impairment During the COVID-19 Pandemic

People with visual impairments may have a higher risk of contagion by SARS-CoV-2 because daily they need to be in contact with objects, surfaces, or assistive devices to recognize the environment and to move in the space. For this reason, the countries around the world focused on recommendations based on frequent handwashing, cleaning of assistive devices, such as sticks, guide dogs, and other recommendations mentioned in Table 2.

### People With Hearing Impairment in COVID-19 Times

Around all continents, the need to implement sign language to obtain better and clearer access to all information about COVID-19 has been recognized. This action would improve the rights of people with hearing impairment. The government states that not only translation is important, but it needs actions to prevent and mitigate the spread of COVID-19 in this population, such as the following measures shown in Table 3.

**Table 3:** Measures for people with hearing impairment in the COVID-19 pandemic.

**Type of measures**	**Description**	**Countries**
Prevention and mitigation of COVID-19	Government information about COVID-19 in sign language. Sign language is regulated for the broadcast of information in the media. Measures on the prevention of contagion and general aspects of the COVID-19 disease (transmission, mechanism of infection, etc.). Aspects related to the quarantine measures and mandatory confinement in the countries. Prevention of contagion through guides, videos, video channels, primers, documents, pictograms, pages, and others Channels or special lines of attention are designed for people with hearing disabilities	Singapore (25), Uruguay (79), Mexico (82), Japan (83), Colombia (84), Argentina (85) Brazil (34), Uruguay (86), Qatar (72), Finland (87), Australia (88) United States (89), Qatar (90) Mexico (91), United States (92), Germany (93), New Zealand (94), Italy (95) Chile (96), Argentina (97)
Health care services	Availability in health services of a person who knows sign language Creation of masks or face shields that allow lip reading Company of an interpreter in health care. The special line to contact the emergency service	Indonesia (41) Chile (98), Canada (24), Singapore (25), Ethiopia (99), United Kingdom (100), Canada (24), France (43)

### People With Physical Impairment in COVID-19 Times

Most of the countries have a general concern regarding the people with physical disabilities, that is the cleaning of assistive devices and surface contact areas because this population usually needs help from another person and close contact with people or their own devices (refer to Table 4).

**Table 4:** Measures for people with physical impairment in the COVID-19 pandemic.

**Type of measures**	**Description**	**Countries**
Health care services	If it is not urgent, try to avoid going to hospitals, physical therapy or activities with shared equipment and using instead tele-rehabilitation services COVID-19 assessment centers must be accessible and adapted for physical disability	Mexico (101) Lithuania (102), Indonesia (41), Canada (22)
Assistive devices	Daily cleaning and disinfection of technical aids and devices (i.e., wheelchairs, walkers, prostheses) Preventing that other people have contact with the cane or the wheels of the wheelchair, cleaning and washing hands more frequently. General maintenance and use of prostheses and orthotics, assistive devices, and artificial limbs	Colombia (21), Chile (103), Brazil (34), Mexico (22), Qatar (104), Uruguay (105), United States (74), India (106), South Africa (107) Colombia (21), Chile (103), Mexico (108) Colombia (21), Mexico (108), Qatar (72)
Exercise in home	Prescription of neck exercises in quarantine. Position changes to prevent pressure ulcers.	India (109)

### People With Impaired Cardiopulmonary Function in COVID-19 Times

Countries focus their attention on preventing contagion by COVID-19 in people with any impaired cardiopulmonary function because they have a higher risk to develop secondary complications due to Coronavirus. In this way, most measures seek to prioritize the special care that this population group should have when they have a virus infection (refer to Table 5).

**Table 5:** Measures for people with impaired cardiopulmonary function during the COVID-19 pandemic.

**Type of measures**	**Description**	**Countries**
Promotion	Encourage a healthy lifestyle in the pandemic, such as: limiting the consumption of foods rich in sodium, avoiding alcohol, tobacco and a sedentary lifestyle, promoting hydration.	Spain (110), Japan (111), Qatar (104), India (112), New Zealand (113)
Prevention	Call to action for the protection of people with cardiopulmonary limitation due to their increased risk of complications associated with COVID-19 infection Strategies to minimize COVID-19 exposure in cardiovascular patients because acute cardiac injury in COVID-19 manifests as left ventricular (LV) dysfunction, heart failure, ventricular arrhythmias, ECG changes, elevated B-type natriuretic peptide (BNP) and troponin Choose disinfectants that are less probability to cause an asthma attack such as products with hydrogen peroxide or ethanol. Limit the use of chemicals that can trigger asthma attacks, such as sodium hypochlorite or quaternary ammonium compounds, and do not use them in enclosed spaces Focus on patient care and avoid leave alone people with chronic or congenital respiratory diseases, bronchiectasis, cystic fibrosis, heart disease, heart failure, valves disease, coronary or congenital heart disease.	Mexico (22), United Kingdom (114), Canada (115), France (116), Qatar (104), Lithuania (117), Puerto Rico (118), Sweden (119), Australia (120), India (112), New Zealand (120), Nigeria (121), Singapore (122), Germany (123), United States (74) Australia (120), New Zealand (120) United States (124), Spain (125), Japan (126) Argentina (127)
Health care services	Prioritizing care for the population with chronic obstructive pulmonary disease, asthma, bronchiectasis, post-tuberculosis, interstitial lung disease, high blood pressure or chronic heart disease, heart surgery, or NYHA stage III or IV heart failure.	United Kingdom (114), Spain (110), Colombia (128), Canada (115)
Adherence to treatment	Continuing drug treatment for any cardiopulmonary health condition. Do not abandon the prescribed medication. Acute myocardial infarction care continues to be carried out as a priority during the pandemic.	Brazil (34), Qatar (104), India (112) Spain (129)
Provision of health services	When it is necessary (cystic fibrosis) to do a respiratory physiotherapy and nebulizations inside the room, it is necessary to closed doors, then ventilate the room Information for patients, their families and caregivers about the right moment to stop physiotherapy for people with neuromuscular disease Encourage differentiation between allergy symptoms so as not to be confused with COVID-19 infection.	Spain (130) Italy (131) Spain (125)

### People With Any Alteration in the Mental Function During COVID-19 Times

Some countries issued information focused on recommendations to orient people with autism, intellectual disabilities, or dementia. Governments issued information in easy-to-read material with information focused on prevention measures to avoid the spread of the virus. Furthermore, countries issued recommendations and advice about the care of mental health in this population during the pandemic (refer to Table 6).

**Table 6:** Measures for people with any alteration in the mental function during the COVID-19 pandemic.

**Type of measures**	**Description**	**Countries**
Prevention and mitigation	Multiple resources for people with autism to continue learning from home and thus prevent infections. Information about COVID-19 pandemic in accessible and understandable material for people with mental disabilities Information about the measures taken globally to prevent contagion, in accessible material. Websites designed for easy access to answer frequently asked questions about everything related to COVID-19. Carry out the COVID-19 test at home for all PWD who need it Guide for health workers about how to explain to people with autism the importance of testing for COVID-19 and mitigating the impact on mental and physical health	Colombia (21) Argentina (83), Singapore (132), Qatar (90), Finland (133), Indonesia (41) Uruguay (134), Mexico (135), Sweden (136) France (137), India (138) Puerto Rico (50) United Kingdom (139), Italy (140)
Education	Accessible material (pictograms, drawings, texts, etc.) is provided to families so that they can explain the current situation to people with intellectual disability. Insistence on the use of biosecurity for people with intellectual disability and those close to them. Use verbal and written reminders constantly explaining the current situation A family calendar is proposed to reorganize daily activities during quarantine Instructions about what to do when a family member of the person with intellectual disability has symptoms of COVID-19. Support or emergency plans are proposed so the person with dementia knows who to turn to or where to call when they need attention	Mexico (141), Spain (142) Canada (24), Argentina (143), Canada (144) Qatar (145), Australia (146), New Zealand (147)
Mental health	Recommendations on the management of panic and anxiety attacks	France (148)
Exercise in home	Physical activity at home during confinement is promoted with exercises designed for PWD. Teaching the caregiver to make video calls and other uses of technology to connect with others. Playful activities for people with autism, to continue in physical activity during quarantine	Chile (149), China (150) India (151) India (152, 153), Indonesia (154)
Exceptions or special considerations	Allowing people with autism and intellectual disabilities to go outside during quarantine, with a family member. Difficulties in understanding biosecurity and prevention measures are recognized, so be flexible with them	Chile (155), Brazil (34)

### Economic and Social Benefits Provided to PWD During the Pandemic

Some American countries, such as Colombia, created programs to identify the PWD who had support needs to ensure their levels of quality of life and food security during the COVID-19 state of emergency (156). Chile made economic donations to PWD (157). Argentina provided economic assistance, such as residences for PWD with the aim of covering expenses for the acquisition of supplies and protection elements directly related to avoiding the COVID-19. Also, they provided financial assistance to the PWD for the acquisition of prophylaxis, prevention, diapers, medicines, and food linked to specific deficiencies or pathologies (158).

Brazil invested in the Social Assistance System with the aim of maintaining programs, projects, and services in the vulnerable areas of the country, mainly for the care of PWD and the elderly (159). The United States earmarked some grants to support PWD and to provide food for the elderly (160).

The European countries, such as France made exceptional provisions to avoid any violation of the rights of the holders of the allowance for PWD, to deal with the social and economic consequences of the COVID-19 epidemic (161). Italy provided bonuses to families to cover the costs of caring for children with disabilities (162). The United Kingdom created some support strategies for PWD, for example, a program of volunteers for the purchase of obtaining essential products and claiming the medicines of PWD. Besides, the PWD received a weekly box of basic supplies, and they were a priority population for deliveries in supermarkets (163).

In Asian countries, such as Japan, there were guidelines for financial support for children with disabilities, due to the temporary closure of special support schools (164). The government of India has a microcredit scheme for PWD to educate or train them in different areas, for their job performance (165); these subsidies were also implemented by other countries, such as Lithuania (102).

In some countries of Oceania, such as Australia, there was a payment to PWD to help them keep their jobs (166). New Zealand indicated what to do if PWD had financial support needs and they created a section with information according to the type of support required, for example, accommodation costs, electricity, gas, water or heating bills, food, school or office costs, and other costs and a guide to help manage money (167).

In Africa, the country of Zambia carried out an inclusive, multi-partner socio-economic impact study on the effects of the COVID-19 outbreak to ensure that no one was left behind by targeting the most vulnerable group, such as PWD and the marginalized groups (168).

## Discussion

In this narrative review, we described the non-pharmacological interventions for PWD that included informative measures and general recommendations during the quarantine period, isolation, and biosecurity measures, contagion prevention, detection of positive cases, mobilization measures, measures implemented in institutions or residences of PWD, the main economic and social benefits provided to PWD, and the measures taken by countries according to the type of impairment (visual, hearing, physical, mental, and cardiopulmonary) during the COVID-19 pandemic.

The WHO called for an action to strengthen the rehabilitation planning and implementation, including sanitary emergency preparedness and response to the current COVID-19 pandemic (8, 169). In this way, the results obtained in this synthesis showed that there has been a response to the COVID- 19 pandemic by 50% of the countries belonging to the United Nations with the aim to protect the health and well-being of PWD, which allows for increasing the visibility of PWD in the society.

The disability considerations of WHO during the COVID-19 outbreak included the following: the provision of accessible information; provision of health services *via* telemedicine and through community-based networks, ensuring equitable healthcare access; guidelines prohibiting blanket decisions on medical rationing, solely on the grounds of disability, employment, and financial protection delivered through disability-related welfare provision; the development of support frameworks for people who need to shield from COVID-19 but who are outside of the social welfare or social care context (e.g., reasonable adjustments in employment working arrangements); education interventions and reasonable accommodations through online special education classes, accessible education activities, and distribution of educational materials; social care services, including psychosocial support, personal assistance, and support for independent living; prevention from and response to violence, in the forms of accessible hotlines for gender-based violence, especially for disabled women, and emergency services and shelters prepared to meet the needs of the disabled people; measures addressing the intersectional disadvantage the disabled people face, including the early release of disabled prisoners, and the provision of accessible health services for homeless people; and the inclusion of disabled people in the recovery phase, ensuring that structural changes are implemented, making the societies more inclusive (8).

Therefore, it is evident that there has been a real concern for the protection of the rights of PWD by supranational organizations which called on governments to guarantee the protection and promotion of the rights of PWD, as evidenced in our recent synthesis about the rights of PWD during the COVID-19 pandemic (18).

Although there have been explicit statements about the prevention and mitigation measures from several countries, others did not do it, as many countries from Africa, Asia, and America especially, central America. Fewer were the declarations to continue with the care of people with rehabilitation needs and the long-term follow-up of PWD. These kinds of responses were timely and more creative on the part of a professional's associations (17). Only 35% of the countries had considerations about employment and financial protection of PWD and accessible education. There were few declarations about the prevention and response to violence, especially for disabled women.

Most of the countries involved in this synthesis had an inclusive response to the pandemic with the creation of measures for PWD but not all countries developed specific measures for each type of impairment. We also note that some countries developed guidelines about the recommendations to be taken for a specific type of disability, while other countries only issued a little information about it; also, some other countries only adapted the recommendations that other countries issued.

The North American countries had a broad focus on the population living in the residential centers or home cares. Some other countries in Europe, such as the United Kingdom and Spain, had a real concern for people with impaired cardiopulmonary function. Most of the recommendations issued by the countries for people with a physical impairment only focused on the cleaning of assistive devices or surface contact areas; however, the recommendations in this population could be covered in a broader way.

However, our findings are consistent with the WHO report that states that PWD may have a greater risk of acquiring COVID-19 because they daily deal with barriers to implementing basic hygiene measures, difficulty in carrying out the social distancing, the need to touch contact surfaces, assistive devices, or some objects to obtain information from the environment, or for the physical support. Besides, some others are institutionalized and others face barriers to the access of the public health information (8).

As Bettger (16) described, the national agencies did not issue specific guidance for the provision of rehabilitation care. According to our synthesis, some countries had a poor response, such as the countries of Africa, in which the information content was less than the other countries of America or Europe; we really do not know if this situation is due to a lack of response or if the measures have not been documented in the official pages. Also, these situations could be explained due to the short time for a COVID-19 response and the quick progress of the current pandemic (16).

Our results are consistent and support Armitage's approach that COVID-19 mitigation strategies must include PWD to ensure that they maintain respect for “Dignity, human rights, and fundamental freedoms, and avoid widening existing disparities” (7). This requires accelerating efforts to include these groups in preparedness and response planning, and requires diligence, creativity, and innovative thinking, to preserve our commitment to the Universal Health Coverage, and ensure that people living with disabilities are not forgotten (7).

Moreover, we share the approach of Ceravolo et al. (170) which stated that the outbreak of the COVID-19 pandemic has challenged the provision of healthcare worldwide, highlighting the main flaws of some health systems concerning their capacity to cope with the needs of frail subjects.

Pandemics, such as COVID-19, place everyone at risk, but certain risks are differentially more severe for groups already vulnerable by the preexisting forms of social injustice and discrimination (10). For this reason, responses to the pandemic must be bound by legal standards, principles of distributive justice, and societal norms of protecting vulnerable populations, core commitments of public health, and to ensure that inequities are not exacerbated and should provide a pathway for improvements to ensure equitable access and treatment in the future (11).

### Limitations

Even though an exhaustive search was made in the national pages of each country, relevant information, issued by different countries, such as reports, guides could have been disregarded due to the language of the information, or perhaps there was no easy way to access this information. The consensus process of recommendations is heterogeneous and, in some cases, not clear. Lack of evidence is inherent because no studies on long-term outcomes were available. Data about the care of PWD in low- and middle-income countries is lacking. There is little information about the measures to continue with the care of people with rehabilitation needs and the long-term follow-up of PWD. Besides, the information is scarce about the consequences of COVID-19 in PWD, and we do not know the effectiveness of the implementation of these prevention measures in disabled people or with rehabilitation needs.

### Implications of the Review

We suggest that appropriate actions and prevention strategies for PWD should be implemented to reduce the contagion of COVID-19, such as vaccination prioritization, access in public places to wash hands when they are away from home, and provision of accessible information for this population through all media.

It is necessary to strengthen and provide health services through telemedicine, telerehabilitation, and home-based rehabilitation, ensuring equitable access to medical care. Besides, we suggest that PWD who need educational interventions and reasonable accommodations for learning can use other methods for their learning, such as online special education classes or accessible educational activities. Also, we consider it important to strengthen social care services for PWD in times of confinement and social distancing.

In those countries where specific guidance for the provision of rehabilitation for PWD was not prioritized, we recommend that national rehabilitation associations and providers make statements to ensure access to rehabilitation services.

Therefore, we call for action from all authorities and other stakeholders to continue and strengthen the creation of measures that manage to address the needs of PWD and to reduce the barriers they experience in the current pandemic. In this contingency, it has not been enough to maintain the health care conditions for PWD. The need to work on strengthening communication and innovation in health care, the integration of countries and human groups to build the way of living in a new world, called the ‘New Normal’ is evident.

## Conclusion

In summary, our findings suggest that most countries around the world have adopted appropriate actions, creating and designing measures and strategies for PWD in response to the health emergency due to COVID-19. The response of different countries to the pandemic showed the need to implement actions for the prevention of contagion of PWD, their families, caregivers, and health professionals who provide rehabilitation services.

However, there is very little specific information available about the measures to continue with the care of people with rehabilitation needs and the long-term follow-up of PWD, and for the prevention and response to violence, especially for women with disabilities. Finally, it is important to highlight that PWD is still a vulnerable population because they are constantly facing barriers that hinder the implementation of prevention and protection measures in the COVID-19 pandemic.

## Author Contributions

LL: definition of the research question and the objectives of the study, elaboration of the methodology for the research, selection of studies, data extraction, synthesis, elaboration of results, and writing the final article. MS: information selection, information research in different organizations, data extraction, information analysis, elaboration of the results, and writing the final article. JV: design and organization of templates for data extraction, content data extraction, and data organization and grouping. MG: analysis and preparation of results and annexes and writing the final article. AP: definition of the research question and the objectives of the study, elaboration of the methodology for the search, selection of studies, and data extraction. VO: synthesis, elaboration of results, selection, extraction, analysis of data from different associations, and writing the final article. LM: selection of studies, data extraction, and synthesis of results. KC: selection of information, information search in different organizations, data extraction, and information analysis. RD: selection, extraction, analysis of data from different associations, and writing the final article. CG: synthesis and elaboration of results and writing and reviewing the final article. DP: data extraction, synthesis and preparation of results, and writing the final article. All authors contributed to the article and approved the submitted version.

## Conflict of Interest

The authors declare that the research was conducted in the absence of any commercial or financial relationships that could be construed as a potential conflict of interest.

## Publisher's Note

All claims expressed in this article are solely those of the authors and do not necessarily represent those of their affiliated organizations, or those of the publisher, the editors and the reviewers. Any product that may be evaluated in this article, or claim that may be made by its manufacturer, is not guaranteed or endorsed by the publisher.
